# Postoperative dysesthesia after PVCR without anterior support applied in Yang's type A severe spinal kyphoscoliosis

**DOI:** 10.3389/fsurg.2023.1222520

**Published:** 2023-07-19

**Authors:** Hangqin Wu, Jie Ding, Junlin Yang, Wenyuan Sui

**Affiliations:** ^1^ Department of Orthopaedic Surgery, Wuyi County First People's Hospital, Zhejiang, China; ^2^ Department of Orthopaedic Surgery, Changxing People's Hospital of Chongming District, Shanghai, China; ^3^ Spine Center, Xinhua Hospital Affiliated to Shanghai Jiao Tong University School of Medicine, Shanghai, China

**Keywords:** postoperative dysesthesia, posterior vertebral column resection, severe spinal deformity, kyphoscoliosis, neurological monitoring

## Abstract

**Backgrounds:**

The incidence and characteristics of postoperative dysesthesia (POD) have not been reported for posterior vertebral column resection (PVCR) in the treatment of severe spinal kyphoscoliosis.

**Objective:**

The objective of the study is to investigate the incidence and characteristics of POD in PVCR without anterior support applied in Yang's type A severe spinal kyphoscoliosis.

**Material and methods:**

From August 2010 to December 2019, 167 patients diagnosed with Yang's type A severe spinal kyphoscoliosis who underwent PVCR without anterior support applied were retrospectively reviewed. All the patients were monitored using five modes of intraoperative multimodal neurophysiological monitoring. Neuromonitoring data, radiographic parameters, and neurological complications were reviewed and analyzed. The incidence and characteristics of POD were further summarized. POD was defined as dysesthetic pain or burning dysesthesia which could be caused by spinal cord kinking or dorsal root ganglion (DRG) injury but with no motor deficits.

**Results:**

PVCR without anterior support was successfully conducted in all 167 patients. Intraoperative monitoring events occurred in five patients. One out of these five patients showed postoperative spinal cord injury (Frankel level C) but completely recovered within 9 months postoperation (Frankel level E). The number of levels and osteotomy space for vertebra resection were 1.28 and 3.6 cm, respectively. POD was confirmed in three patients (3/167, 1.8%), characterized as kyphosis with the apex vertebrae in T12 with the kyphotic Cobb angles of 100°, 115°, and 122°, respectively. The osteotomy space of vertebra resection in these three patients were 3.9, 3.8, and 4.2 cm, respectively. After the treatment by drug administration, they reported pain relief for 12–36 days. The pain gradually moved to the distal end of a proper DRG innervated region near the end.

**Conclusions:**

In this study, the incidence rate of POD in Yang's type A severe spinal kyphoscoliosis patients who underwent PVCR without anterior support applied was 1.8% (3/167). Evoked potential monitoring could not detect the occurrence of POD. POD in Yang's type A severe spinal kyphoscoliosis after PVCR could be ascribed to spinal cord kinking and DRG injury.

## Introduction

In 2016, Yang et al. first reported on a proposed classification system to guide surgical strategy in severe spinal deformity based on spinal cord function (1). The classification system defined type A as patients with normal spinal cord and evoked potentials and no neurological symptoms. Posterior vertebral column resection (PVCR) without anterior support applied was routinely used in our center to treat Yang's type A severe kyphoscoliosis, and similar clinical outcomes were observed as those in previous studies (2–4).

Dysesthesia is commonly reported in diabetes, Guillain–Barre syndrome, Lyme disease, multiple sclerosis, percutaneous endoscopic lumbar discectomy, and oral surgery patients (5–8). Dorsal root ganglion (DRG) injury–related postoperative dysesthesia (POD) has been reported in 0.9% patients with minimally invasive transforaminal lumbar interbody fusion, less than 5% patients with percutaneous endoscopic lumbar discectomy, and 7.7% patients with lumbar spine osteotomies (9–11). Although PVCR-related neurological deficit risks have been repeatedly emphasized (3, 12, 13), the POD has not been reported for PVCR in the treatment of severe spinal kyphoscoliosis (9–11), especially for Yang's type A kyphoscoliosis. Thus, the purpose of this study was to investigate the incidence and clinical characteristics of POD in our institution and present the outcomes of these patients.

## Materials and methods

### Patients

From August 2010 to December 2019, 167 consecutive Yang's type A kyphoscoliosis patients treated with PVCR without anterior support were retrospectively reviewed. The inclusion criteria included (1) scoliosis curve >80° or kyphosis >80°; (2) complete medical record data including radiography, intraoperative neurological monitoring, and spinal cord function; and (3) deformity diagnosed as type A according to Yang's spinal cord classification (normal spinal cord and evoked potentials, no neurological symptoms).

### Radiographic evaluation

Standing anteroposterior and lateral radiographs of the entire spine were conducted before and after surgery. Left and right lateral flexion radiographs, whole-spine computed tomography (CT), and magnetic resonance imaging (MRI) were also performed before surgery. The radiographic variables evaluated in this study were the Cobb angle of major curve and its corrective rate, flexibility, and angle of kyphosis and its corrective rate.

### Surgical technique

The indications for PVCR in the current patients included (1) severe scoliosis (>80°), (2) severe angular or tuberculous scoliosis (>80°), and (3) severity based on quantitative strategy on deformity angular ratio (14). The same senior doctor (JY) performed all surgeries under neurological monitoring. General anesthesia administration and endotracheal intubation were first performed, and then, a skin incision was made over the spinous processes to expose the intended fusion levels. Following pedicle screw placement using the free-hand technique, provisional instrumentation was then placed at the concave side using pedicle screws above and below the level of resection prior to the corpectomy and discectomy. Osteotomies were performed at the apex of kyphosis to increase the effectiveness of correction. Laminectomy was performed to expose the spinal cord. The corresponding rib heads were removed to expose the lateral walls of the pedicles. Then, compression force was applied to shorten the spinal column, and the osteotomy site was closed gradually. A wide laminectomy is often performed to extend one level above and below the PVCR level for better orientation and safety during compression. This approach can prevent shortening of the osteotomy space, which might result in kinking and bulking of the spinal cord. Under neuromonitoring, the osteotomy site must be completely closed so that the bony endplates could contact with each other. More attention should be given to prevent excessive shortening and interference with the intervertebral foramen, especially during osteotomy and correction. The osteotomy distance at the convex lateral border of apical vertebra was then measured and recorded.

### POD

POD was defined as dysesthetic pain or burning dysesthesia which could be caused by spinal cord kinking or DRG injury but with no motor deficits. The visual analog scale/score (VAS) was assessed in patients with POD. Non-steroidal anti-inflammatory drugs (NSAIDs), central non-opioid analgesic agents (tramadol), and neuropathic pain medications (pregabalin) were selectively used to treat POD based on VAS.

### Statistical analysis

Statistical analysis was performed using SPSS version 19.0 for Windows (SPSS Inc., Chicago, IL, USA). The normality of continuous data was determined using the Shapiro–Wilk test. Analysis of variance was performed to compare parameters among three vertebral column resection (VCR) locations. A *P*-value less than 0.05 was considered significant. Data were presented as mean ± standard deviation.

## Results

### Patients

A total of 167 patients (99 males and 68 females), who met the inclusion criteria and received PVCR without anterior support, were included in the analysis. All patients were successfully followed up, with an average duration of 5 years. The mean age at the time of surgery was 25.7 years. The etiological diagnoses included 96 idiopathic cases, 51 congenital cases, and 20 neurofibromatosis cases. Table 1 summarizes the preoperative demographics.

**Table 1:** The patient's preoperative demographics.

Number of the patients	167
Male/female	99/68
Mean age (years)	25.7 (range: 11–54)
Diagnosis	96 IS, 51 CS, 20 NF
Halo gravity traction	21
Instrument	All pedicle screw system
Mean follow-up (months)	60 (range: 1–120)

IS, idiopathic scoliosis; CS, congenital scoliosis; NF, neurofibromatosis.

### Content of PVCR surgery

**Figure 1:**
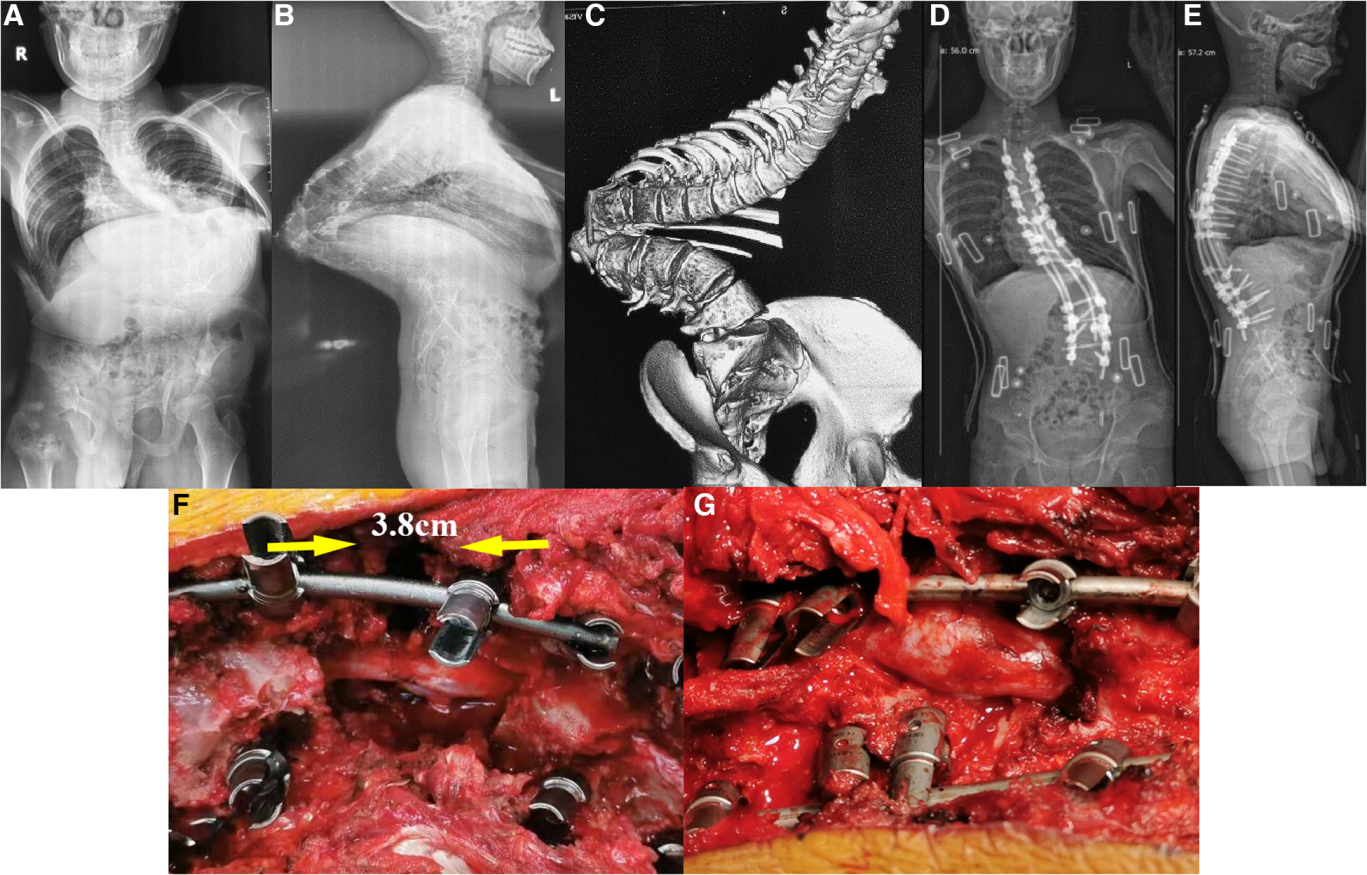
A 21-year-old male with congenital scoliosis with Yang's type A kyphoscoliosis treated with PVCR without anterior support. The patient was diagnosed with congenital scoliosis with the apex located at T12 preoperatively. (**A–C**) The preoperative X-ray and CT of the patient; (**D,E**) postoperative X-ray of the patient; (**F,G**) state of the spinal cord before and after the closure of the osteotomy gap during the operation. It can be clearly seen that there are bulking/kinking in the spinal cord after the closure of the osteotomy gap.

**Figure 2:**
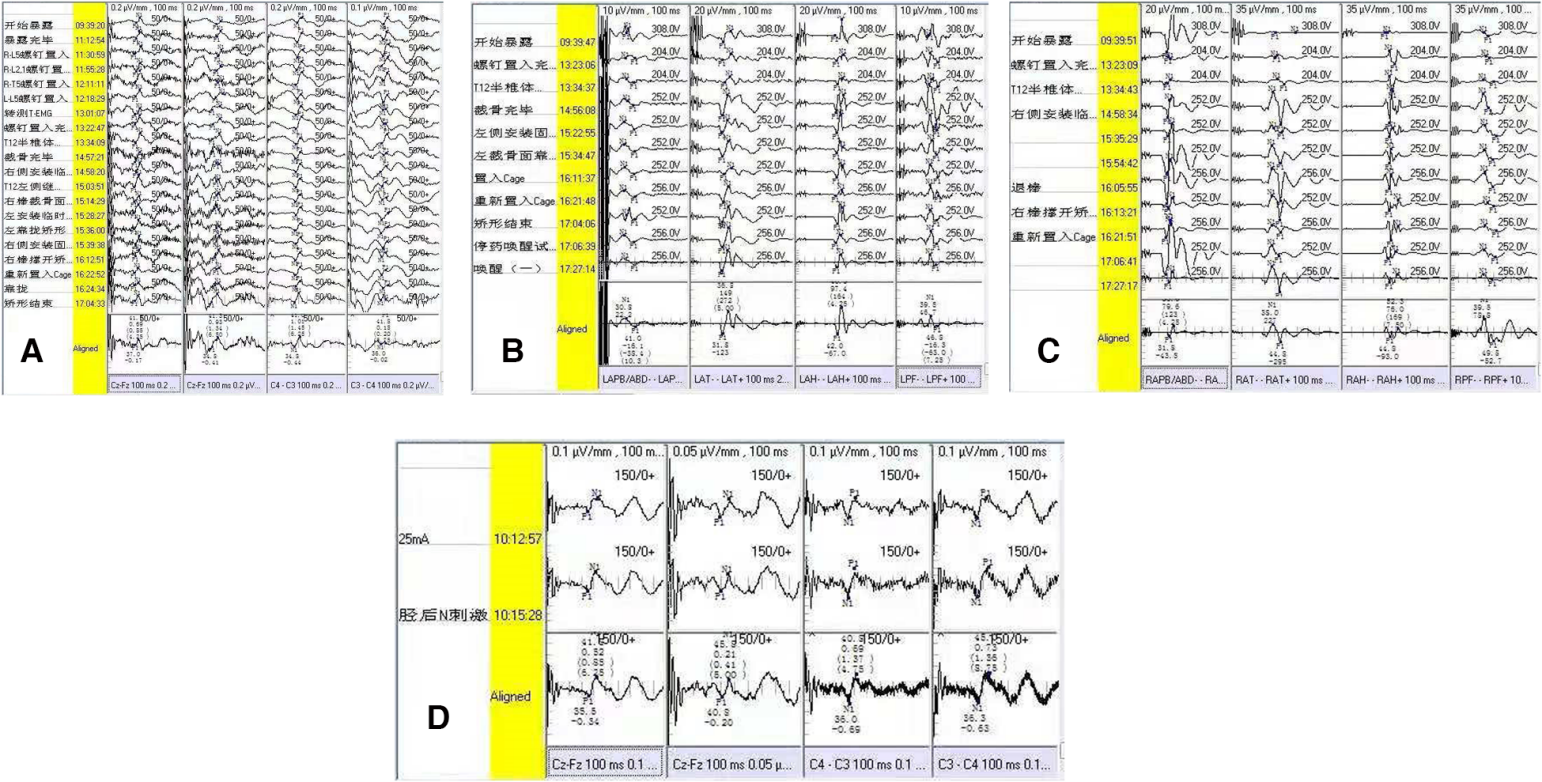
Neuroelectrophysiological monitoring outcome of the patient with postoperative dysesthesia. (**A**) Intraoperative SEP monitoring for bilateral lower extremities; (**B**) intraoperative left-MEP monitoring; (**C**) intraoperative right-MEP monitoring; (**D**) postoperative SEP monitoring 3 day later. There were no intraoperative evoked potential changes (**A–D**).

No structural anterior support was applied in all 167 PVCR patients (Figure 1). The number of levels for vertebra resection was 1.3 (range: 1–2 levels), with an average osteotomy distance of 3.6 cm (range: 2.7–5.0 cm) (Figure 2). There were 145 one-level VCRs and 22 two-level contiguous VCRs. According to the location of VCR, there were 22 high thoracic cases (T1–T4) with 3.6 ± 0.9 cm osteotomy distance, 112 middle thoracic cases (T5–T8) with 3.8 ± 1.2 cm osteotomy distance, 33 lower thoracic cases (T9–T12) with 3.9 ± 0.7 cm osteotomy distance. The average intraoperative blood loss and surgery time were 2,831.2 ± 965.4 ml and 479.2 ± 68.3 min, respectively. There were no significant statistical differences in the number of levels for vertebra resection, osteotomy distance, blood loss, and surgery time among the three VCR locations (*P*-value > 0.05).

### Scoliosis and kyphosis correction rates

The preoperative coronal plane major curve (with a mean flexibility of 17.6%) was corrected from 126.4 ± 28.3° to 55.0 ± 16.5°, showing a mean of 58.2% correction. Preoperative thoracic kyphosis was corrected from 128.4 ± 24.3° to 53.3 ± 16.5°, showing a mean of 62.1% correction. There were no significant statistical differences in all these radiographic parameters among the three VCR locations (*P*-value > 0.05) (Table 2 and Figure 1).

**Table 2:** Radiographic parameters and intraoperative condition.

Parameters	Value
Coronal major curve	
Pre-op (°)	126.4
Flexibility (%)	17.6%
Post-op (°)	55.0
Corrective rate (%)	58.2%
Sagittal kyphosis	
Pre-op (°)	128.4
Post-op (°)	53.3
Corrective rate (%)	62.1%
Osteotomy numbers	1.28 (range: 1–2)
Osteotomy distance (cm)	3.6 (range: 2.7–5.0)
Intraoperative blood loss (ml)	2,831
Surgery time (min)	479

### Intraoperative and postoperative neural event

In this study, intraoperative monitoring events occurred in five patients (5/167). One out of the five patients showed transient spinal cord injury (a decrease from Frankel level E to Frankel level C) after the operation, whose cord function recovered to Frankel level E after 9 months of follow-up.

### POD

Immediately after operation, three cases of POD were confirmed (3/167, 1.8%), which were characterized as kyphosis with the apex vertebrae in T12, with kyphotic Cobb angles of 100°, 115°, and 122°, respectively. The osteotomy space of vertebra resection in these three patients were 3.9, 3.8, and 4.2 cm, respectively. After the patients woke up from anesthesia, POD was characterized by persistent lateral or bilateral lower limb hyperalgesia without motor impairment. The degree of pain was intense, with a VAS score of more than seven points. Slight skin touch can induce or aggravate the degree of pain. After treatment by drug administration (NSAIDs, tramadol, or pregabalin), all three patients reported pain relief with a duration of 12–36 days (Table 3). We routinely adopted five evoked potential monitoring modes, and no intraoperative evoked potential changes occurred in the three patients. Somatosensory evoked potential (SEP) was re-monitored for these patients 3 days after operation, and the results were also normal, indicating that the occurrence of POD could not be detected by neuroelectrophysiological monitoring (Figure 2). In the process of rehabilitation, the POD region of three patients gradually moved to the distal lower extremity, which finally disappeared. None complained of any symptoms of POD at the final follow-up. Thus, there was a favorable prognosis for POD.

**Table 3:** Details of the three POD cases.

Patient	Age	Gender	Diagnosis	Scoliosis Cobb angle (°)	Kyphotic Cobb angle (°)	Apex vertebrae	Osteotomy space (cm)	Dural bulking/kinking
1	18	M	Idiopathic	100	120	T12	3.9	Y
2	21	M	Congenital	118	125	T12	3.8	Y
3	17	M	Congenital	110	135	T12	4.2	Y

## Discussion

This study found that the incidence rate of POD in Yang's type A severe spinal kyphoscoliosis patients who underwent PVCR without anterior support applied was 1.8% (3/167). Although the occurrence of POD is not associated with motor dysfunction, its impact on the patients’ quality of life is quite significant and should be taken seriously by surgeons.

It has been reported that intraoperative DRG injury was responsible for POD, and it was caused by intraoperative pulling of nerve tissues, mechanical stimulation, bipolar electrical coagulation disturbance, and accumulation of closed spinal cord in osteotomy (15, 16). In our series, 1.8% (3 out of 167) patients underwent POD, while all of them were characterized as severe kyphosis with apex vertebrae in T12. The osteotomy space of vertebra resection in the three patients were 3.9, 3.8, and 4.2 cm. Dural kinking and bucking due to the bone-on-bone technique after PVCR were obvious in these three patients. Thus, we speculated that, in addition to related causes reported in literature, the magnitude of kyphosis angle, kyphosis apex position, osteotomy closure distance, and spinal cord kinking and bulking might be the main risk factors for POD in PVCR surgery in the treatment of Yang's type A severe spinal kyphoscoliosis.

The main manifestation of POD in this study was hyperalgesia in the lateral or bilateral lower limbs, with severe pain (VAS scores > 7), which is more serious than that of previous investigations (9–11). The pain can be induced or aggravated by slight skin touch, similar to the characteristics in published literatures (17, 18). However, all the three patients showed no lower limb motor dysfunction, and the manifestations of their nervous system were mainly hyperalgesia. The deep reflex, deep sensation, and pathology of lower limbs were negative. One patient initially presented with impaired left lower limb movement caused by severe pain (VAS 9), and the pain relief by analgesic drugs (celecoxib and pregabalin) proved that motor function was barrier-free.

There is no unified standard for the diagnosis of POD, which mainly depends on clinical symptoms and clinical experiences (19, 20). In our series, two patients (2/3) showed dysesthesia in the lateral or bilateral limbs, one patient showed anaphylaxis in the L1 and L2 nerve root innervation regions, and another showed anaphylaxis in the L3 and L4 regions without motor dysfunction. Another patient (1/3) showed hyperalgesia in areas L4 and L5 combined with left lower limb extensor knee movement disorder. After the use of analgesics, motor dysfunction was confirmed to be caused by pain. POD is easily misdiagnosed as nerve root injury, making it essential to exercise caution when diagnosing POD, especially when the nerve injury and POD occur at the same time (21, 22). Our experience is that isolated POD cannot be diagnosed until motor dysfunction is completely excluded. We routinely adopted five evoked potential monitoring modes throughout the whole operation, and no intraoperative evoked potential changes occurred in the three patients. We re-monitored the three patients with SEP 3 days after operation, and the results were still normal, indicating that the occurrence of POD could not be recognized by neuroelectrophysiological monitoring. In all the three patients, the pain gradually moved to the distal end of a proper DRG innervated region during the recovering period.

The treatment of POD aimed to relieve pain and shorten the recovery period. NSAIDS, opioids, glucocorticoid drugs, antidepressants, and neuralgia drugs have been reported to show certain clinical effects (9–11). All the three patients presented initial VAS scores of more than seven, and favorable outcomes were obtained with the treatment of pregabalin combined with celecoxib. The pain of three patients was relieved by about 50% the first day after medication treatment, and the lower limb pain of three patients was completely relieved with medication treatment for 12–36 days. Previous literature recommended antidepressant drugs for the treatment of POD (23). In addition, intervertebral foramen block, with analgesic drug and steroid administration, also has been shown effective (24, 25). However, we did not use this treatment strategy in this study because the combination of celecoxib and pregabalin already achieved significant pain relief.

Although it has been reported that the prognosis of POD after routine spinal surgery is generally satisfactory, the prognosis of POD after PVCR in severe spinal kyphoscoliosis has not been well described. According to the condition of our three patients, the paresthesia could be completely relieved after 12–36 days of treatment, which is similar to previous studies (23–25). In addition, we believe that the gradual migration of the anaphylaxis plane to the distal extremity during the treatment process could be a significant feature indicating the potential for a cure for POD.

This study has several limitations. First, this was a retrospective study performed in a single center by a single surgeon using one technique. Therefore, it is challenging to generalize the incidence and prognosis of POD to other patients and other techniques of PVCR. In addition, there were insufficient patients (three patients) diagnosed with POD in the current study, so the symptom, pathogenic mechanism, and treatment strategy should be further investigated and discussed.

In conclusion, in the current study, the incidence of POD in Yang's type A severe spinal kyphoscoliosis patients who underwent PVCR without anterior support applied was 1.8% (3/167). Evoked potential monitoring could not detect the occurrence of POD. POD in Yang's type A severe spinal kyphoscoliosis after PVCR could be ascribed to spinal cord kinking and DRG injury.

## Data Availability

The raw data supporting the conclusions of this article will be made available by the authors, without undue reservation.
